# CLL Exosomes Modulate the Transcriptome and Behaviour of Recipient Stromal Cells and Are Selectively Enriched in miR-202-3p

**DOI:** 10.1371/journal.pone.0141429

**Published:** 2015-10-28

**Authors:** Mosavar Farahani, Carlos Rubbi, Luning Liu, Joseph R. Slupsky, Nagesh Kalakonda

**Affiliations:** 1 Department of Molecular and Clinical Cancer Medicine, Institute of Translational Medicine, University of Liverpool, Liverpool, United Kingdom; 2 Department of Functional and Comparative Genomics, Institute of Integrative Biology, University of Liverpool, Liverpool, United Kingdom; 3 Department of Haematology, Royal Liverpool University Hospital, Liverpool, United Kingdom; University of Manitoba, CANADA

## Abstract

Bi-directional communication with the microenvironment is essential for homing and survival of cancer cells with implications for disease biology and behaviour. In chronic lymphocytic leukemia (CLL), the role of the microenvironment on malignant cell behaviour is well described. However, how CLL cells engage and recruit nurturing cells is poorly characterised. Here we demonstrate that CLL cells secrete exosomes that are nanovesicles originating from the fusion of multivesicular bodies with the plasma membrane, to shuttle proteins, lipids, microRNAs (miR) and mRNAs to recipient cells. We characterise and confirm the size (50–100 nm) and identity of the CLL-derived exosomes by Electron microscopy (EM), Atomic force microscopy (AFM), flow cytometry and western blotting using both exosome- and CLL-specific markers. Incubation of CLL-exosomes, derived either from cell culture supernatants or from patient plasma, with human stromal cells shows that they are readily taken up into endosomes, and induce expression of genes such as c-fos and ATM as well as enhance proliferation of recipient HS-5 cells. Furthermore, we show that CLL exosomes encapsulate abundant small RNAs and are enriched in certain miRs and specifically hsa-miR-202-3p. We suggest that such specific packaging of miR-202-3p into exosomes results in enhanced expression of ‘suppressor of fused’ (Sufu), a Hedgehog (Hh) signalling intermediate, in the parental CLL cells. Thus, our data show that CLL cells secrete exosomes that alter the transcriptome and behaviour of recipient cells. Such communication with microenvironment is likely to have an important role in CLL disease biology.

## Introduction

Chronic lymphocytic leukemia (CLL) is characterised by accumulation of monoclonal mature B-lymphocytes in the circulation and tissues.[1, 2] The malignant lymphocytes depend on micro-environmental cues and factors for accumulation and survival.[3, 4] A myriad of factors that support CLL cell growth and proliferation are described including secreted cytokines such as IL6, IL21, and IL4, cell-contact elements such as CD40-CD154, and integrin-ligand interactions.[3] These reports have mainly addressed the effects of the microenvironment on the phenotype of CLL cells. However, whether CLL cells can affect the behaviour and phenotype of supportive cells within the stromal microenvironment is not widely addressed.

Cellular communication typically involves secreted factors and direct cell contact. Recent studies have demonstrated an additional layer of intercellular communication involving the secretion and uptake of extracellular vesicles (EVs).[5] Exosomes are a discrete population of small (50–100 nm diameter) EVs of endosomal origin with a lipid membrane bilayer and a cup-shaped morphology.[6] Exosomes encapsulate selected membrane and cytoplasmic proteins and can influence the phenotype and behaviour of adjacent or distant cells through the transfer of messenger and microRNAs (mRNA and miRs).[5, 7–9] Exosomes derived from mouse mast cells are shown to deliver mRNA to human mast cells with the subsequent expression of murine proteins within the human recipient cells.[10] Successive studies demonstrate similar exosome-mediated transfer of mRNA and miRs to other cells of the immune system, including B cells, in order to modulate behaviour. Similarly, tumour derived exosomes modulate the microenvironment to promote disease progression in glioblastoma[11] and other cancers.[12–14]

With respect to CLL, microvesicles derived from the malignant cells in this disease are shown to transfer the phospho-receptor tyrosine kinase Axl to stromal cells to create a “homing and nurturing” environment.[15] Recent work has demonstrated the presence of miR-155 in microvesicles derived from the plasma of CLL patients with progressive disease.[16] This is pertinent as miRs are critical for CLL pathogenesis and deregulated expression of miRs, such as miR-155, segregates with aggressive phenotypes and poor prognosis.[17–19] So far, direct transfer of CLL-derived miRs to cells in the microenvironment cells has not been demonstrated.

Given that secreted exosomes facilitate intercellular communication and signalling, we investigated the physical and functional characteristics of these vesicles released by CLL cells. We explored the hypothesis that uptake of CLL derived exosomes can lead to reprogramming of the transcriptional profile of recipient cells. Towards this end, we isolated exosomes from CLL cell cultures and patient plasma. Characterisation of these exosomes showed that their physical properties are consistent with those described for such EVs derived in other systems. Examination of the effects of CLL-derived exosomes on human stromal cell behaviour showed that these EVs perturb gene expression within, and enhance proliferation of, target recipient stromal cells. Analysis of their miR cargo showed that CLL-derived exosomes have a unique signature that is not merely a reflection of the miR content of the parental cells. More importantly, we demonstrate a selective enrichment of miR-202-3p in CLL exosomes. Thus, this study a) provides evidence for exosome mediated communication of CLL cells with the microenvironment and b) identifies a unique exosomal molecular content that is relevant in disease biology.

## Materials and Methods

### Patient samples, cell lines, and cell culture

The collection and use of the primary patient material used in this study had approval from the Liverpool Research Ethics committee. Primary mononuclear (CLL and normal B) cells from fresh or viably frozen samples were purified by MACS isolation as previously described.[50] B-cell purity (>95%) was assessed by flow cytometry. Only samples from untreated CLL patients were included in this study (Table A in S1 File). CLL cells (2 x 10^7^ cells/ml) were cultured in RPMI-1640 supplemented with 1% Bovine Serum Albumin (BSA), 2 mM L-glutamine, 100 U/ml penicillin, and 100 μg/ml streptomycin (Invitrogen, UK). BSA was used to avoid potential contamination with exosomes in foetal calf serum. MEC1 (CLL derived line)[51] and HS-5 (a human BM cell line)[52] were cultured in RPMI medium.

### Isolation of exosomes

CLL cells were cultured for 48h and media supernatant was harvested. Exosomes were purified from this supernatant using the scheme illustrated (Figure A in S1 File), following the procedure described by Thery et al.[20] with minor modifications. Following the final wash step, the resultant pellet containing exosomes was resuspended in 30–50 μl PBS (or appropriate lysis buffer), protein content was determined by Bradford assay and the suspension stored at -80°C until used. An alternative method of isolating exosomes was also employed. This involved incubating culture supernatants overnight with anti-HLA magnetic beads, and then capturing the bound exosomes using a magnet (Dynal Biotech).[53]

### Phenotypic and physical characterisation of exosomes

For surface protein expression analysis, exosomes prepared by ultracentrifugation were adsorbed on to aldehyde-sulphate latex beads (3.8μm) (Molecular probes, Invitrogen) as described.[20] Here 10μg of exosomes in 100μl PBS were incubated with 25μl bead suspension overnight. The beads were washed, stained with antibodies of interest, and finally analysed by flow cytometry (FACS Calibur; BD Biosciences).[54] For analysis by Western blotting, exosome-associated proteins (5–10μg) solubilised in SDS-PAGE buffer were separated by electrophoresis on polyacrylamide gels, and transferred to an immobilon solid support for chemiluminescent protein detection. The antibodies used are listed in Table B in S1 File. Further physical characterization of isolated exosomes was achieved by two independent methods:

#### Transmission Electron microscopy

Exosome pellets isolated by ultracentrifugation were fixed in 2% paraformaldehyde and adsorbed on to Formvar-carbon coated grids. These grids were then fixed with 1% glutaraldehyde, contrasted in 2% uranyloxalate- / uranylacetate-methylcellulose solution and air-dried. When exosomes isolated by magnetic beads were used, these were fixed in 2.5% (v/v) glutaraldehyde for 2 hrs before embedding them in 4% agar. The exosome-agar blocks were fixed with 1% osmium tetroxide (45 mins), and then stained with 2% Uranylacetate (in 0.69% maleic acid). The blocks were dehydrated with washes of solution with ascending ethanol concentration, and then embedded in epoxy resin (TAAB) for polymerisation (at 60˚C overnight). Ultrathin sections (90nm) were cut from this final preparation on a Reichert-Jung ultramicrotome and stained with Reynold’s lead citrate (5 mins). Images were viewed in a transmission electron microscope (Philips EM208S).

#### Atomic Force Microscopy (AFM)

Five μg of purified exosomes in de-ionized H_2_O were added to a freshly cleaved mica sheet and incubated for 10 min. The sheet was rinsed with de-ionized H_2_O, and air-dried. A Multimode 8 atomic force microscope (Bruker), equipped with a 160-μm J-scanner and oxide-sharpened Si_3_N_4_ cantilevers (600μm, *k* = 0.4 N·m^−1^) was operated in peak force tapping mode for imaging. Topographic height, DMT (Derjaguin-Muller-Toporov) modulus and adhesion images were recorded simultaneously at 512×512 pixels (scan rate = 1Hz). Image processing required NanoScope Analysis software (Bruker).

### Exosome-labelling and immunofluorescence microscopy

Exosomes were fluorescently labelled using the PKH67 dye (Sigma-Aldrich) as previously described.[33] Staining efficiency determined by FACS analysis was >90%. For uptake studies, PKH67-labelled exosomes were seeded, in serum free media, on to a sub-confluent layer of HS-5 (5x10^5^) cells adherent to a cover slip within a 24-well plate. Free PKH67 in diluent served as control. After 2-hour incubation with exosomes or free dye, the culture media was changed and cells allowed to proliferate overnight. Cells were then harvested, fixed with 4% paraformaldehyde, permeabilised with a 0.1% solution of Triton X-100, stained with primary antibody of interest, followed by a fluorescent-tagged secondary antibody and counterstained with DAPI. Resultant cell preparations were examined using an Olympus BX61 microscope (100X magnification). Images were captured with a Photometrics Cool Snap HQ2 camera and MicroManager Software and were analyzed with Image J software (NIH).

### RNA isolation, cDNA preparation, miR array analysis, RT-qPCR, and miR transfections

Total RNA extractions were performed using the miRNeasy kit (Qiagen), and quantitatively and qualitatively assessed using a Nanodrop ND-1000 (Nanodrop) and a Bio-analyser 2100 (Agilent). For miR expression analysis, fluorescence-labelled exosomal RNA (180 ng) and total cellular RNA (1μg) samples were submitted for data acquisition (Exiqon miRCURY^TM^ LNA array v.5) and analysis (background subtraction and normalisation).

Reverse transcribed microRNA was prepared using a miScript II kit (Qiagen), and subjected to RT-qPCR analysis using commercial primers (Qiagen) and miScript SYBR Green PCR Kit. Analysis was carried out on an Mx3000P machine (Stratagene). miR expression was calculated either by the standard curve method, or determined relative to RNU6B using the 2^–ΔΔCt^ method. Sufu and GAPDH primer sequences for mRNA expression analysis by RT-qPCR are listed in Figure B in S1 File.

The HS-5 cell line was transfected with miR-202-3p mimic or scrambled control (Life technologies) using the Lipofectamine 2000 transfection reagent according to the manufacturer’s protocol (Invitrogen). Cells were harvested for analysis after 24h.

Gene expression analysis of HS-5 cells incubated with exosomes was performed using the Human Pathway Finder PCR array (Qiagen). Data analysis was performed using the software provided (Qiagen). Fold changes in gene expression were calculated using GAPDH normalisation and the 2^–ΔΔCt^ method. Results were verified independently by RT-qPCR. HS-5 cells ‘spiked’ with exosomes served as controls.

### Assay for c-fos activity

Nuclear c-fos activity was quantified using a TransAM^®^ AP-1/c-fos ELISA assay kit (Active motif) following the supplied protocol and a Gemini Fluorescence microplate reader (450 nm) (Molecular Devices).

### Cell proliferation

Relative cell numbers were assessed, after generation of a standard curve, using the CyQuant NF cell proliferation assay kit (Invitrogen), following manufacturer’s instructions. Values were normalised to untreated controls.

## Results

### Tumour derived exosomes exhibit physical characteristics that reflect the derivation from parental CLL cells

Exosomes were isolated from cell culture supernatants after a series of ultracentrifugation steps (Figure A in S1 File) as previously described.[20] Method optimisation was achieved using culture media from two B-cell lines [Raji (B-cell lymphoma) and MEC-1 (CLL derived)], and applied to culture supernatants of freshly isolated CLL cells (purity and viability >95%) that were cultured for 48h in RPMI-1640 medium supplemented with 1% BSA but not serum to avoid potential contamination by exogenous exosomes. We next characterised these CLL cell-derived exosomes for their phenotypic and physical properties.

**Fig 1:**
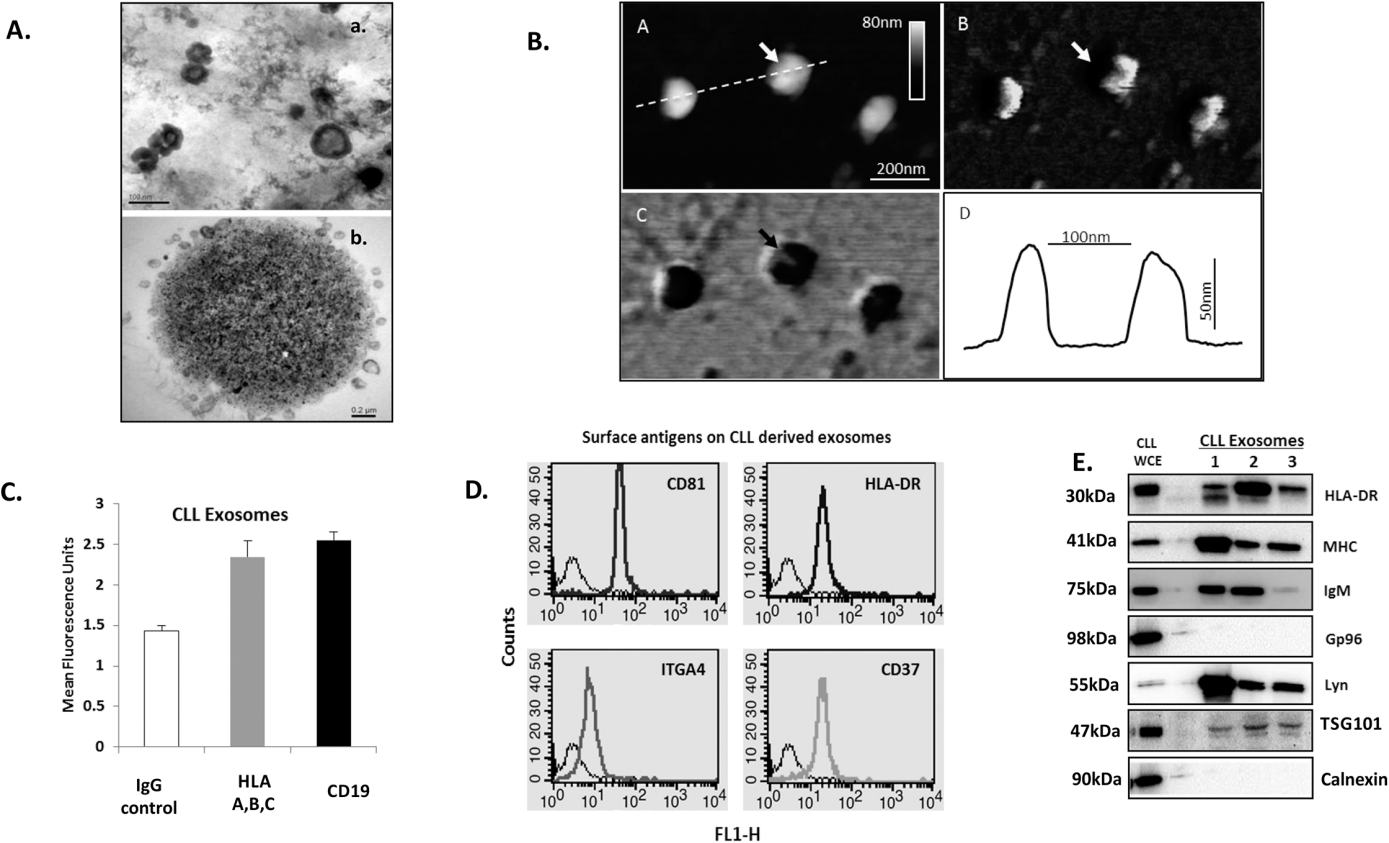
Characterization of CLL derived exosomes. **A)** Transmission electron microscopy (TEM) image of exosomes enriched from CLL cell culture medium after 48 hrs. (a) Exosomes are visible as small 50–100 nm vesicles with bi-layered membranes (scale bar: 100 nm). (b) Exosomes were immune-isolated using magnetic beads coated with anti-HLA antibody and ultrathin sections of resultant beads processed for image analysis. The representative TEM image shows a bead coated with exosomes (scale bar: 0.2**μ**m). **B**) AFM images of exosomes from a representative CLL case immobilized on a mica surface using Peakforce Tapping mode (Multimode 8, Bruker). A) Topographic image of exosomes. **B**) DMT Modulus image C) Adhesion image: The exosomes appear as circular biconvex vesicular structures. The DMT Modulus and adhesion images of exosomes show explicit sub-structures at the centre of the vesicles (arrows). D) Schematic cross-section following the line in the indicated exosome in C). **C)** FACS analysis of B-cell antigens on CLL exosomes prepared from primary CLL cases by density ultracentrifugation and adsorbed onto 4**μ**m aldehyde-sulphate latex beads and incubated with isotype control, anti-HLA-A, B, C or CD19 antibodies followed by FITC-conjugated secondary antibody. Mean fluorescence intensities are plotted (mean ± standard error of mean (S.E.M.) (n = 9)). **D)** Representative analysis showing surface expression of CD81, HLA-DR, CD37, and integrin α4 (ITGA4) in CLL derived exosomes coupled to aldehyde-sulphate latex beads. Binding of FITC-conjugated isotype controls are included for comparison. **E)** Immunoblot analysis of CLL derived exosomes: Lysates from CLL exosome were probed for abundance of HLA-DR, MHC, IgM, Gp96, Lyn kinase, TSG 101 and Calnexin. Images are representative of analyses of 3 cases. *WCE-Whole cell extract*.

To investigate the size and typical morphological characteristics of the isolated exosomes, we undertook transmission electron microscopy (TEM) analysis. Fig 1A **(**upper panel) shows that the harvested CLL exosomes derived using our ultracentrifugation protocol have typical contours (cup-shaped morphology) and size (50–100 nm) consistent with an exosome-specific phenotype. For additional confirmation, we then employed a second method of isolation in which CLL exosomes were purified using anti-HLA magnetic beads. Here, TEM analysis of ultrathin sections of agar embedded beads showed that the exosomes had similar morphological characteristics to those isolated by ultracentrifugation (Fig 1A, lower panel). Finally, atomic force microscopy (AFM) was used to generate three-dimensional images for more detailed morphological analyses of the exosomes in their native state. Fig 1B shows confirmation of the biconvex spherical structure that is characteristic of exosomes, and of their diameter (93.5 ± 11.6 nm) and height [74.8 ± 11.2 nm (n = 30)] dimensions.

Exosomes exhibit surface characteristics that reflect their cells of origin.[21] To examine the characteristics of CLL-derived exosomes, we initially adsorbed them on to aldehyde sulphate latex beads and then labelled with antibodies directed against CD19 and MHC class I for analysis by flow cytometry. Fig 1C demonstrates that exosomes derived from primary CLL samples (n = 9) show enhanced expression of these B-cell markers. Further analysis of these exosomes, using a broader panel of antibodies, showed that they also express HLA-DR (MHC class II) and the tetraspanins CD81 and CD37 (Fig 1D). Such antigenic expression is characteristic of both exosomes in general and of B-cells.[22] In a specific instance, where the CLL cells expressed ITGA4, the secreted exosomes also expressed this integrin on their surface (Fig 1D, lower left panel). The CLL-derived exosomes were then subjected to Western blot analysis (Fig 1E). Here we confirmed expression of TSG-101, MHC classes I and II, IgM, and Lyn kinase. Notably, the endosplasmic reticulum (ER) associated proteins Gp96 and Calnexin were absent, confirming that the CLL-derived exosomes were not contaminated with other membrane-derived structures, but encapsulate proteins that are characteristic of both the donor cells and of multivesicular bodies in general (MVB).[23]

In summary, we demonstrate that CLL cells produce vesicles whose physical and morphological characteristics reflect their cellular origin and an exosomal phenotype.

### CLL-derived exosomes are internalised by stromal cells and localise to late endosomes

**Fig 2:**
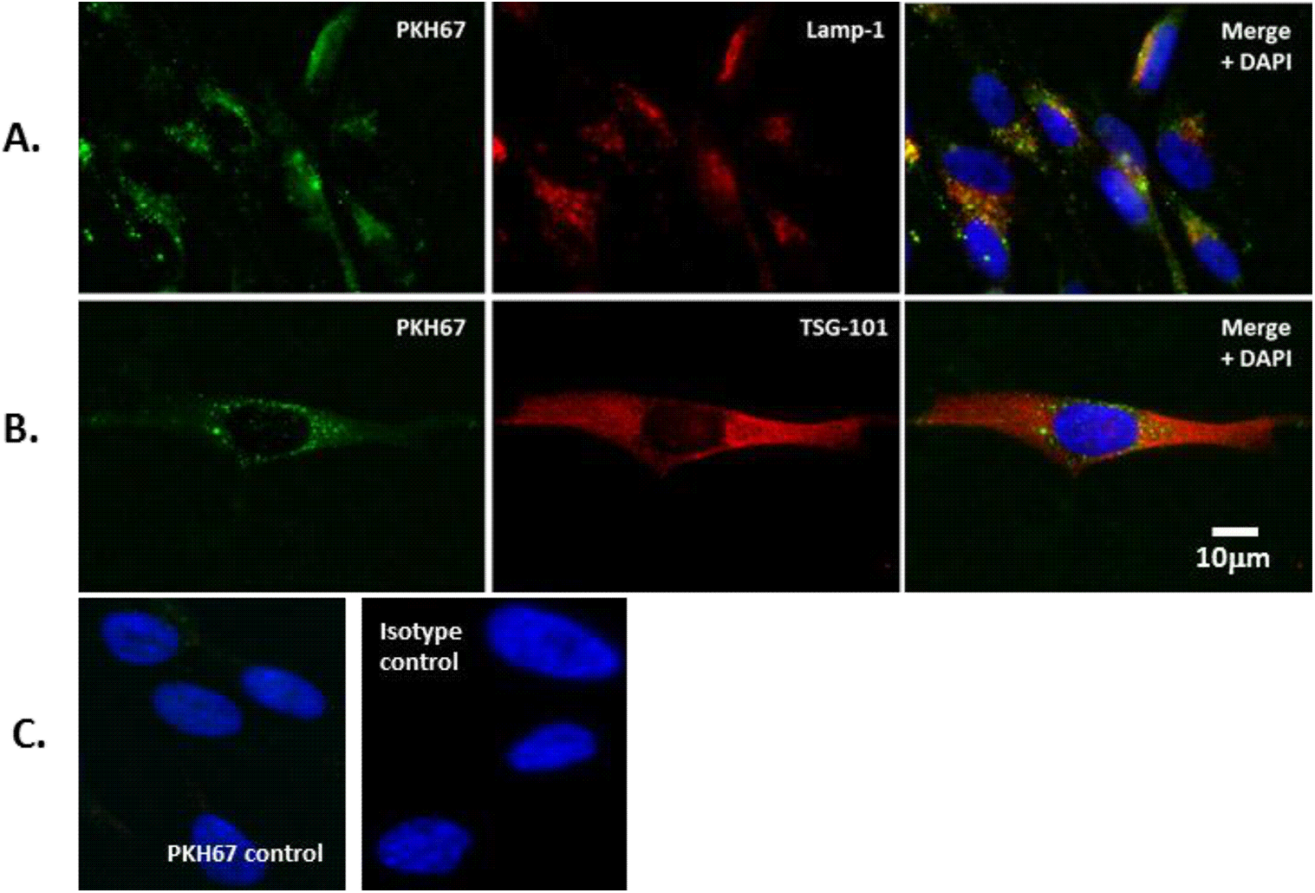
CLL-derived exosomes are internalised by stromal cells and localise to late endosomes. Exosomes purified from CLL cell culture supernatants were labelled with the lipophilic dye PKH67. HS-5 stromal cells were cultured in the absence (control) or presence of fluorescent-labelled exosomes for 24hrs. Paraformaldehyde fixed HS-5 cells were permeabilised and stained for Lamp-1 or TSG-101, followed by FITC-conjugated secondary antibody and visualised by fluorescence microscopy (original magnification, ×100). Nuclei were stained using DAPI. Internalised exosomes co-localise with Lamp-1, a marker for late endosomes but not with TSG-101. A) Exosomes were internalised by HS-5 Cells and visualized for PKH67 (green), Lamp-1 (red) and DAPI (blue). Co-localisation of Lamp-1 and PKH67 appear in yellow. B) HS-5 Cells were visualized for PKH67 (green), TSG-101 (red) and DAPI (blue) and do not show co-localisation C) As controls. HS-5 cells were stained with free PKH67 dye without exosomes or antibody isotype control to exclude non-specific binding. (Scale bars: 10**μ**m).

Intercellular communication by exosomes is accomplished by the uptake of these vesicles into recipient cells by processes such as endocytosis,[24, 25] resulting in the transfer of their molecular content and subsequent modulation of recipient cell behaviour.[12, 26] To model CLL cell interaction with the microenvironment we used the normal human bone marrow stromal cell line HS-5. We labelled purified exosomes from primary CLL (n = 3 cases) cell culture medium with a green fluorescent lipophilic dye (PKH67) as previously described.[24] These labelled exosomes were added to adherent HS-5 cultures in serum-free media, and after 2-hour incubation at 37°C the cells were washed, and then further cultured overnight with the addition of fresh medium. Next, the HS-5 cells were fixed, permeabilised, and stained with antibodies that recognise either TSG-101 (a marker of the endosomal sorting complex (ESCRT) required for transport), or Lamp-1 (a marker of late endosomes) [24, 27], and analysed by fluorescence microscopy (Fig 2). HS-5 cells incubated with PKH67-labelled CLL exosomes showed a distinct patchy intracellular green fluorescence pattern within their cytosol, a pattern of staining that was not present in the control HS-5 cells exposed to an equivalent amount of free PKH67 dye (lower left panel, Fig 2). This suggests that the HS-5 cells internalise the labelled exosomes. In co-localization studies, we observed that the internalised exosomes co-associated with Lamp-1, but not TSG101 (right hand panels), suggesting that the exosomes were transported to endosomal organelles within the HS-5 cells. Similar results were obtained for exosomes sourced from the MEC-1 cell line culture medium (not shown). Taken together, these results show intracellular sorting of internalised exosomes by HS-5. PKH67-labelled exosomes are trafficked to late endosomes as confirmed by co-localization of PKH67and Lamp-1 in treated HS-5 cells.

### Uptake of CLL exosomes, derived from in vitro and in vivo sources, by recipient stromal cells has functional consequences and promotes cell proliferation

**Fig 3:**
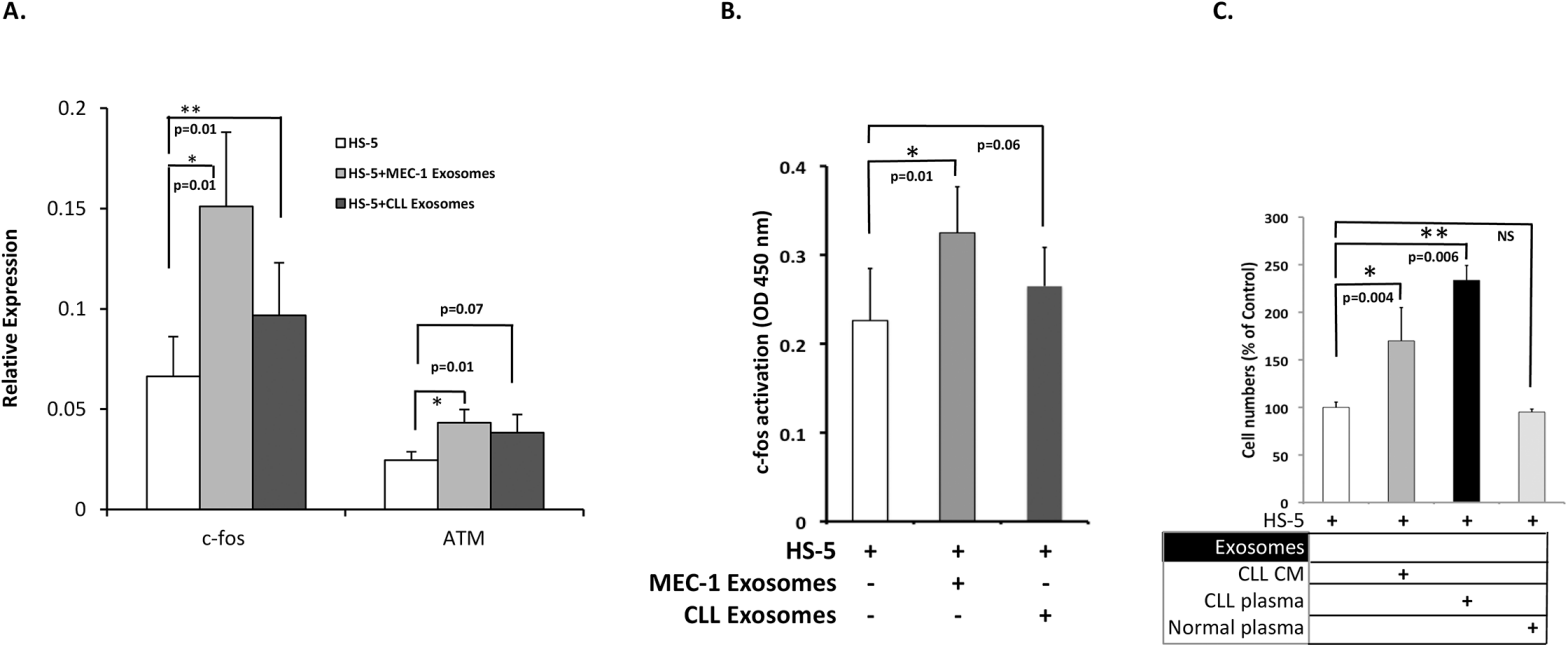
CLL derived exosomes modulate the gene expression profiles of HS-5 cells and alter the rate of their proliferation. **(A)** HS-5 cells were treated with MEC-1 exosomes for 24hrs and a pathway array analysis of cellular RNA was performed (S2 File). Untreated HS-5 cells ‘spiked’ with equivalent doses of MEC-1 exosomes served as control. Validation of c-fos and ATM expression level changes determined by additional RT-qPCR is shown. Error bars represent ± S.E.M. of three independent experiments. *p* values indicating level of significance are shown. **(B)** Exosomes influence c-fos activation in recipient cells: c-fos heterodimer complex DNA binding activity was detected using an ELISA transcription factor assay kit (Active Motif). HS-5 cells were cultured alone or with exosomes as indicated. Nuclear extracts from MEC-1 and CLL exosome treated HS-5 cells show increased c-fos activation (p = 0.009 and p = 0.06 respectively). **(C)** HS-5 cells plated at a density of 3000 cells/well were incubated in the absence or presence of exosomes derived from primary CLL cell culture medium (CLL CM), CLL patient plasma or normal healthy donor plasma, (150ug/ml) and proliferation measured after 48 hrs using the CyQuant assay. Paired t-test was used to calculate p values. All data represent mean ± S.E.M of triplicate experiments.

Release of exosomal contents, into recipient cells should lead to altered gene expression and result in modulation of cellular behaviour. To study the consequences of exosomal uptake, we incubated HS-5 cells with MEC-1 derived exosomes and addressed the effects on gene expression patterns of recipient stromal cells. In this experiment, we used an RT^2^ Profiler PCR array (see methods) to detect alterations in expression of 84 selected genes critical for pathways such as cell proliferation, apoptosis, and the cell cycle in exosome-treated and control HS-5 cells. Among the genes differentially expressed, ATM (3.1-fold), c-fos (2.36-fold), ITGAV (2.05-fold), TNFRSF25 (2.25-fold) and PNN (2.92 fold) were overexpressed, while E2F1 (-1.45) and BAD (-1.25) were downregulated in exosome-treated HS-5 cells. The expression of the selected genes in treated and untreated HS-5 cells and fold changes in all 84 genes are detailed in S2 File. Additionally, we sought to confirm the upregulated expression of c-fos and ATM by independent RT-qPCR analysis of cDNA from exosome-treated cells compared to untreated cells ‘spiked’ with an equivalent amount of exosomes. As shown in Fig 3A, HS-5 cells incubated with exosomes derived from cell culture supernatants of both MEC-1 cells and primary CLL cells (n = 3) significantly upregulated c-fos expression (p = 0–01). Although there was a similar increase in ATM levels in HS-5 cells on exosomal incubation and uptake from both sources, MEC-1 derived exosomes were relatively better at upregulating the expression (p = 0.01 vs 0.07). As exosome-spiked HS-5 cells were used as control, this confirms that altered expression of these genes was not merely due to passive transfer of exosomal mRNA content. To further examine the functional consequence of c-fos gene overexpression, HS-5 cells were treated for 24 hrs with exosomes isolated from MEC-1 or CLL cells for 24hrs. Nuclear extracts were assessed by an ELISA based c-fos activation assay (Fig 3B). Compared to untreated HS-5 cells, MEC-1 exosome treated cells showed a significant increase in c-fos activation (p = 0.01). Although not quite reaching significance, a similar trend (p = 0.06) was also noted for cells treated with exosomes derived from primary CLL cells. These results indicate that exosome uptake actively affects the transcriptome of stromal cells with functional consequences.

The importance of direct interactions between CLL cells and stromal cells for tumour cell survival and proliferation is well documented.[28] The contribution and importance of exosomes, and their cargo, in inter-cellular communication is less clear. To address the physiological relevance of our findings above, we sought to examine the effects of CLL exosomes on stromal cell proliferation. Fig 3C shows that HS-5 cells cultured in the presence of CLL-derived exosomes showed enhanced proliferation in comparison with control cells (p = 0.004).

We next compared the effects of exosomes derived from the plasma of CLL patients with that from normal subjects (n = 3 each). In keeping with the result obtained with *in vitro* derived exosomes, *in vivo* sourced CLL exosomes positively influenced stromal cell proliferation (Fig 3C, p = 0.006) whereas exosomes derived from normal plasma had no effect.

In conclusion, our studies provide proof that CLL cells secrete exosomes, both *in vitro* and *in vivo*, and that these exosomes have comparable effects on stromal cell proliferation. As several studies have proposed that transfer of exosomal microRNA may modulate the biological function of the recipient cells [29], [30], [14], [31], we next investigated the microRNA cargo of these CLL derived exosomes.

### CLL exosomes selectively encapsulate miRs and are enriched in miR-202-3p

Previous studies have shown that exosomes encapsulate functional mRNAs, miRs and proteins that reflect their cell of origin [5, 10, 32, 33]. Given the importance of miRs in CLL pathobiology [17, 19, 34], we hypothesised that CLL derived exosomes should contain unique miRs that are reflective of CLL cell content and additionally relevant for disease biology. To this end, total RNA was extracted from paired exosomal preparations and parental cells from six CLL cases. Initial analysis of this extracted RNA showed that exosomes were more abundant in small RNAs compared to total cellular RNA, and also lacked 18S and 28S ribosomal RNA (Figure C in S1 File). We next performed parallel miR expression profiling of exosomal (180 ng) and total cellular RNA (1μg) using an LNA array platform (consisting of 899 human miRs) as detailed in the Supplementary section (S3 File).

**Fig 4:**
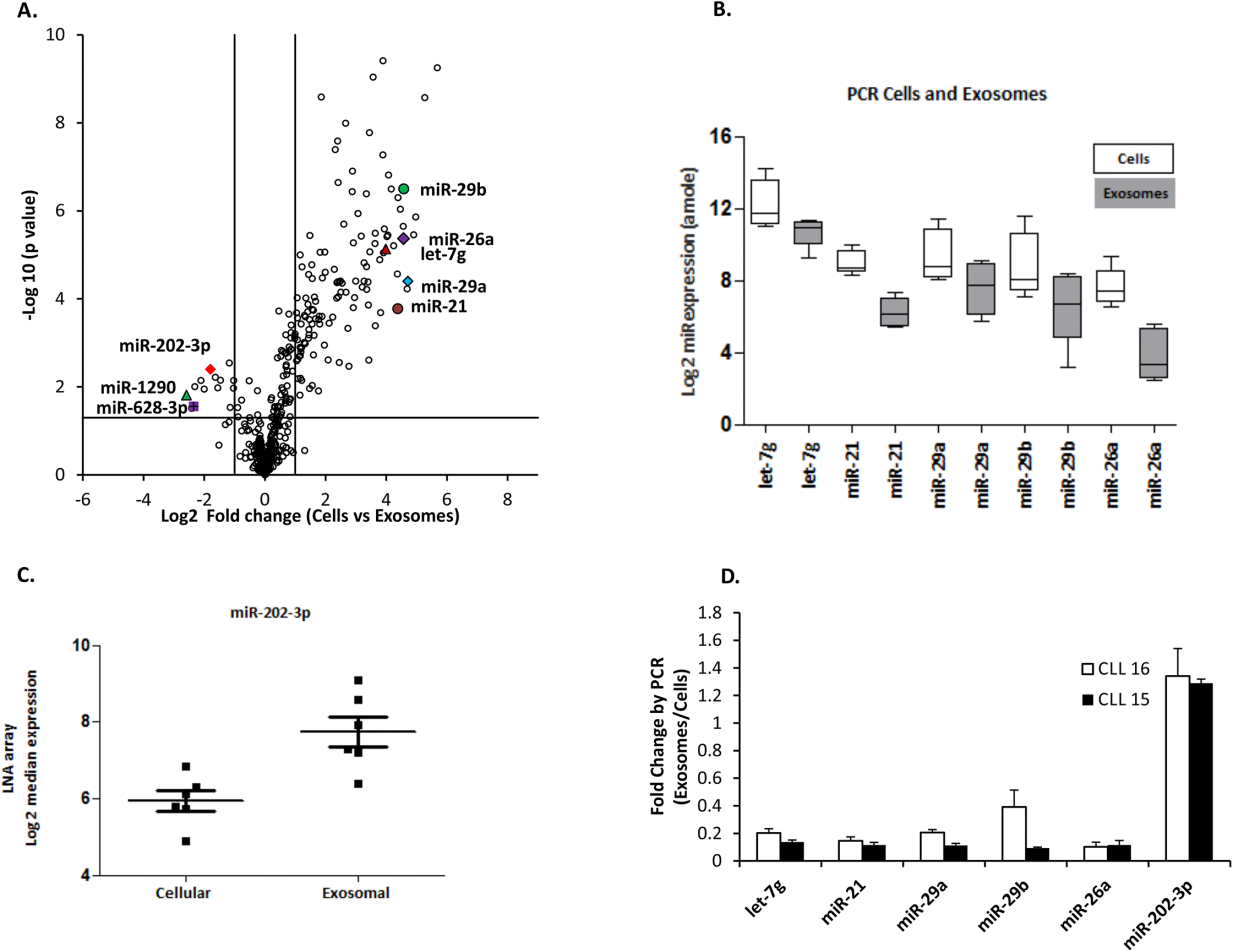
MicroRNA profiles and RT-qPCR of purified exosomes and parental CLL cells. **A)** A volcano plot of LNA array miRNA profiles of CLL cells vs exosomes samples is depicted. The x-axis shows the Log2 fold-change in miRNA expression between cellular cases and exosomes. The y-axis shows the -Log10 of the p value for each miRNA. Expressed miRs that are statistically significant between the two groups appear above the line (p < 0.05). **B)** The miRs let-7g, miR-21, miR-29a, miR-29b, and miR-26a were selected for validation of LNA array by RT-qPCR analysis (n = 5). Data represent mean ± S.E.M of triplicate experiments. **C)** miR-202-3p is enriched in exosomes compared to parental cells in the LNA array data (mean ± S.E.M). **D)** Fold change of miR expression in exosomes vs cellular CLL samples showing the enrichment of miR-202 by RT-qPCR in two representative cases.

In order to visualize the differences between the two sources we analysed fold differences and generated a volcano plot. In general, the miR content of the exosomes reflected the content in the parental cells with enrichment of miRs that are relevant in CLL cell biology. However, we found that certain candidate miRs are specifically enriched or counter-selected in exosomes including miR-202-3p, miR-1290, and miR-628-3p but are less abundant in the parental cells (Fig 4A).

To validate these findings of the LNA array, we performed RT-qPCR analysis of CLL cells and the derived exosomes for all samples (Fig 4B). Due to the lack of a suitable miR shared by both cellular and exosomal samples that lends itself as an optimal normalisation control, measurement of miRs with the RT-qPCR protocol was performed using the mirVana^TM^ miRNA Reference Panel v9.1 (Ambion, UK) which allows quantitation based on the generation of standard curves generated for specific miR sequences. We selected miR-29a, miR-29b, miR-26a, and let-7g as these species are highly expressed in CLL cells and are relevant for disease biology [35]. Importantly, miR-1290, miR-628-3p, and miR-202-3p were enriched within the exosomal RNA (Fig 4A). As high levels of miR-202-3p were seen in all six exosomes samples compared to parental CLL cells (Fig 4C) in the LNA array, we focused on miR-202-3p for further studies. A further analysis of miR-202-3p expression in paired exosomal RNA and parental cells by RT-qPCR confirmed significant enrichment of miR-202-3p in contrast to other miRs that are equally abundant in cells and exosomes. Fig 4D shows fold change in specific miR expression in exosomes vs. cells in two representatives CLL cases. Taken together, these data show that CLL exosomes carry a miR cargo, which largely represents their cell of origin, but are also selectively enriched for miR-202-3p.

### CLL exosomes alter the levels of miR-202-3p and its target Sufu mRNA in recipient cells

**Fig 5:**
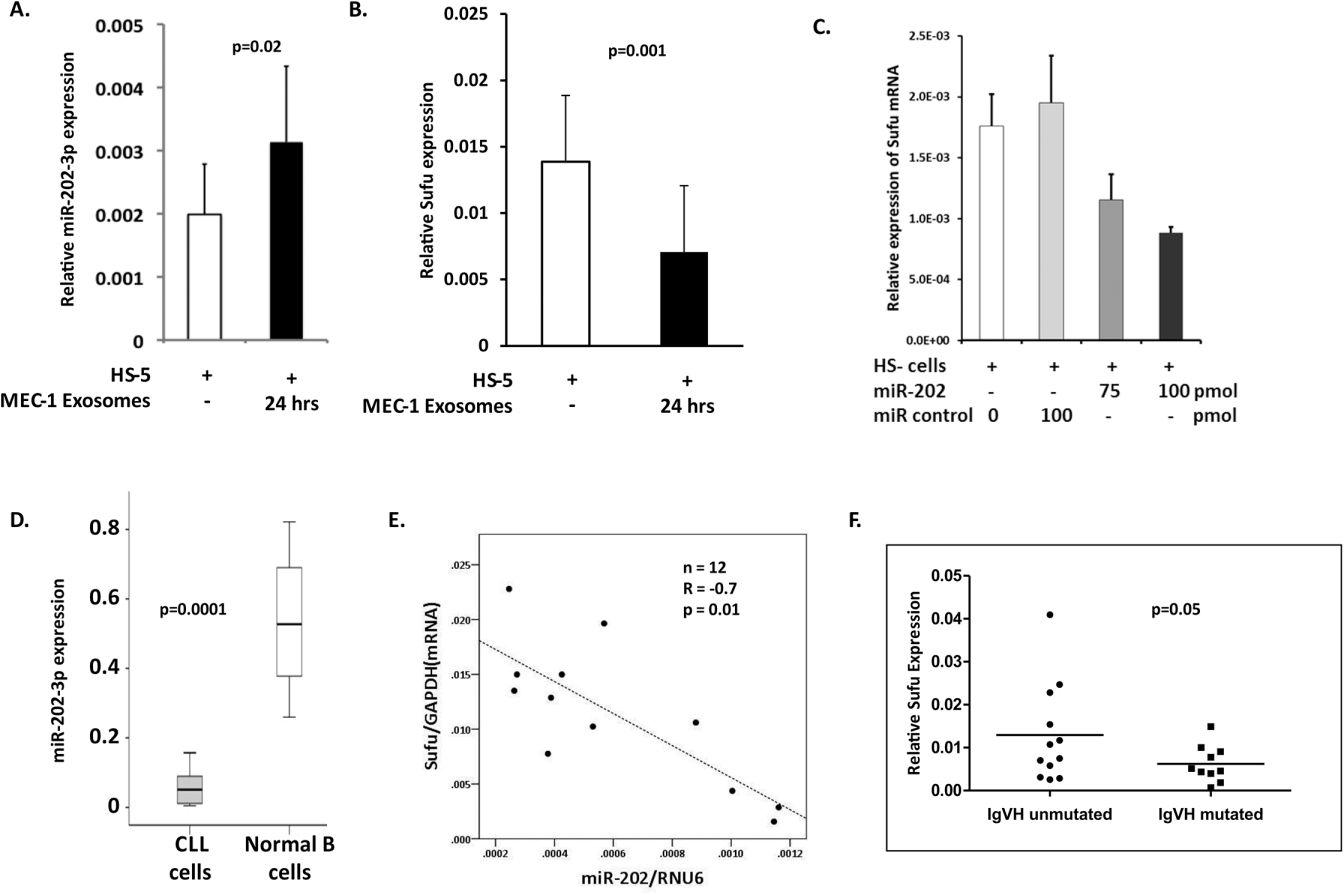
Expression of miR-202-3p and its target Sufu mRNA in CLL cells. **(A)** HS-5 cells incubated with MEC-1 exosomes for 24 hrs show increased expression of miR-202-3p by RT-qPCR analysis. The results represent the findings of three independent experiments. **(B)** HS-5 cells incubated with MEC-1 exosomes for 24 hrs show decreased expression of Sufu, one of the miR-202-3p targets, by RT-qPCR analysis. The bar graphs are derived from three independent experiments. **(C)** RNA extracted from HS-5 cells transfected overnight with a synthetic miR-202-3p mimic or scrambled control, was subjected to RT-qPCR to assess Sufu mRNA expression. **(D)** RT-qPCR of miR-202-3p, using RNA prepared from CLL cells (n = 19) or normal B cells (n = 4). Box plots show the expression level of miR-202-3p relative to a miR-U6B control for normalisation. **(E)** Primary CLL cell lysates (n = 12) were subjected to RT-qPCR analysis for miR-202-3p and Sufu mRNA expression levels, normalised to RNU6 and GAPDH expression respectively, and show statistically significant inverse correlation. Plotted data represent mean of experiments performed in triplicate. **(F)** Sufu mRNA expression, using RT-qPCR and GAPDH as reference, in primary CLL cells of 12 unmutated and 10 mutated CLL cases is shown. Data demonstrate relatively higher (p = 0.05) levels of Sufu expression in the IgVH unmutated cohort. The expression in individual cases represents the mean of triplicate measurements.

To study whether encapsulated miRs in CLL exosomes are transferred to recipient cells we incubated HS-5 cells with MEC-1 derived exosomes, as miR-202-3p expression levels in exosomes derived from MEC-1 cells or primary CLL cells are quantitatively similar (not shown). Fig 5A shows that such incubation results in a statistically significant increase in miR-202-3p levels (p = 0.02) in treated HS-5 cells compared to untreated controls. Next, we focused on a known target of miR-202-3p, namely Sufu a regulator of the Gli-Hedgehog signalling pathway with an established role in the oncogenesis of CLL.[36] Our selection was based on interrogation of the miRWalk database for miRNA-target interactions.[37, 38] In fact, both Sufu mRNA transcript variants 1 and 2 contain miR-202 target sequences in their 3’ untranslated region (UTR) (Figure D in S1 File).

We show that in HS-5 cells treated with MEC-1 exosomes, there is a significant reduction in Sufu mRNA expression (Fig 5B; p = 0.001). Our PCR primers were designed to amplify both variants of Sufu mRNA. This data supports an effect of exosomal miR-202-3p on target HS-5 cellular Sufu mRNA.

Indeed, HS-5 cells tranfected with a miR-202-3p mimic, but not a scrambled oligonucleotide control, showed a concentration-dependent reduction in Sufu mRNA levels (Fig 5C). In addition, there was a corresponding decrease in Sufu protein levels on transfection of HS-5 cells with the miR-202-3p mimic compared to the scrambled control (Figure E in S1 File). To understand the relevance of miR-202-3p expression in CLL, we performed an RT-qPCR analysis of cellular RNA in CLL cells, and compared this with that in normal B-lymphocytes. Fig 5D shows that CLL cells contain significantly lower levels of miR-202-3p compared to normal B-cells. We next compared miR-202-3p levels in exosomes derived from CLL cells (n = 9) and normal B-cells (n = 3) and found variable expression by RT-qPCR analysis. The exosomes from normal B-cells do contain slightly higher levels of miR-202-3p compared to the CLL cohort (Figure F in S1 File) and likely reflect the higher levels in normal B-cells compared to CLL cells as noted above (Fig 5D). This would suggest that in contrast to normal B-cells, there is a significant active and deliberate packaging of miR-202-3p into CLL exosomes. Furthermore, miR-202-3p expression showed a significant negative correlation with Sufu mRNA levels in CLL cells (Fig 5E; R = -0.70, p = 0.01). These data support linked tumour-suppressive and oncogenic roles in CLL for miR-202-3p and Sufu respectively. To evaluate the relevance of Sufu expression in disease biology, we subjected primary cell lysates from 12 IgVH unmutated and 10 mutated CLL cases to RT-qPCR analysis for Sufu mRNA expression using GAPDH mRNA for reference. IgVH umutated CLL cases, with reported poorer outcomes, have relatively higher levels of Sufu expression (Fig 5F; p = 0.05).

### Expression of selected miRs in exosomes derived from CLL patient plasma

**Fig 6:**
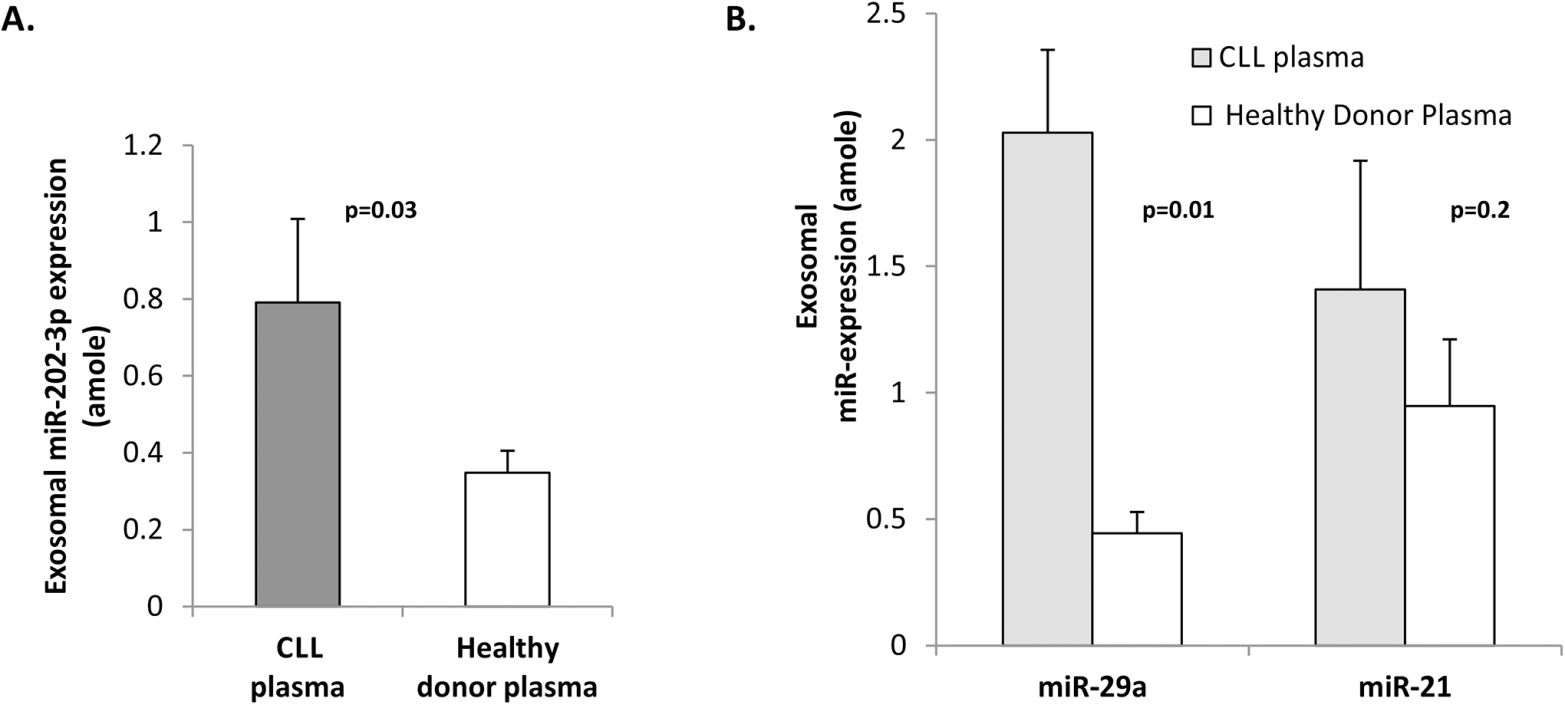
Expression of miRs 202-3p, 29a, and 21 in exosomes derived from plasma of CLL patients versus normal donors. **(A)** Exosomes sourced from plasma obtained from CLL patients or healthy donors (n = 3 each) were subjected to RT-qPCR analysis for absolute levels of miR-202-3p using a standard curve method. The levels of miR-202-3p are significantly higher in exosomes purified from CLL plasma (p = 0.03). **(B)** Absolute levels of miR-29a and miR-21 in exosomes harvested from CLL patient and healthy donor plasma (n = 3) were determined by RT-qPCR analysis. Standard curves for miR-29a and miR-21 were generated using the miRVana^TM^ miRNA reference panel v9.1. Statistical analysis was performed using an unpaired t-test and yielded *p values* of 0.01 and 0.2 for miR-29a and miR-21 respectively.

To obtain confirmation of selective enrichment miR levels in exosomes derived from in vivo sources, we compared the expression of miR-202-3p in exosomes derived from CLL and healthy donor plasma by RT-qPCR analysis and show that there are significantly lower levels in the latter (Fig 6A; p = 0.03, n = 3 cases). This was also evident for miR-29a and miR-21 which were relatively higher in CLL plasma derived exosomes compared to those sourced from healthy donor plasma (Fig 6B; n = 3 each). This suggests the existence of active regulatory pathways that lead to higher expression of selected miRs in CLL exosomes and plasma and may have utility as biomarkers of disease behaviour.

In conclusion, the relatively higher levels of miR-202-3p in CLL derived exosomes, compared to the parental cells suggests that such selective sequestration into the secreted exosomes is an active and regulated process, and of likely relevance in disease biology and behaviour.

## Discussion

In recent years, the versatile role of exosomes in cell-cell communication, in physiological and pathological states, has attracted a great deal of attention and is of particular relevance in cancer.[6, 21, 39, 40] Exosomes shuttle molecules such as proteins, mRNAs and miRs between cancer cells and their microenvironment to influence disease behaviour. In this study we aimed to characterise exosomes secreted by CLL cells, examine their potential effects on the microenvironment, as well as explore the nature of their miR content.

Our data provide a physical description of exosomes derived from CLL cells. We have purified these secreted vesicles from supernatants of cultured CLL cells using established protocols [20] and show that their size (50–100 nm) and morphology are consistent with that of exosomes derived from other cell systems. [23] Phenotypic characterisation confirmed that CLL cell-derived exosomes carry classical exosomal markers such as MHC class I and II molecules, CD81, and TSG-101, as well as elements unique to CLL cells such as IgM, CD19, Lyn, and the integrin ITGA4 in a specific instance. Our work can be distinguished from the findings of Ghosh et al [15] who isolated, and functionally characterised CLL microvesicles, which differ with respect to their biogenesis and physical characteristics. Whereas microvesicles (100–1000 nm) form by budding from plasma membranes, exosomes are derived from intracellular endosomes that fuse to the plasma membrane for release, implying a more active process.[6] Hence, we chose to focus our efforts on isolation of exosomes that are likely distinct in their molecular content and function due to their mode of origin.

Earlier studies of microvesicles and exosomes have demonstrated that these vesicles transfer RNA and protein content to alter recipient cell behaviour.[10, 11, 15] In line with other studies,[24, 25] we demonstrate that CLL exosomes are taken up by stromal cells and localise to late endosomes in the recipient cells as evidenced by co-localisation with the specific marker Lamp-1. It is notable that, as with microvesicles, CLL exosomes actively influence the stromal cell transcriptome and alter their growth characteristics. Melo et al.[31], show that primary breast cancer exosomes initiate non-cancerous epithelial cells to form tumours by rapid silencing of target mRNAs to reprogram the target cell transcriptional profile. We show that CLL exosomes similarly modify the gene expression profiles of stromal cells. CLL exosomes significantly increased c-fos and ATM levels in stromal cells that was not merely a reflection of passive transfer of exosomal mRNAs. c-fos functions as a subunit of a group of dimeric transcription factors collectively referred to as activating protein 1 (AP-1) that are implicated in a wide range of processes, including proliferation and oncogenic transformation.[41] Hence, we studied the effects of CLL exosomes derived from CLL culture medium and patient plasma on proliferation of HS-5 cells. We show that CLL exosomes significantly increase HS-5 proliferation compared to those derived from normal plasma. This is in line with a previous study by Gutzeit et al., who showed that exosomes from Burkitt’s lymphoma cell lines enhance proliferation in recipient cells.[26]

Secretion of exosomes from B-cell lines is well documented but their release from normal or malignant primary B-cells is less well studied.[42–44] The generation of exosomes is not a constitutive activity of normal B cells, but is induced following activation signals such as IL-4 and anti-CD40.[45, 46] For our studies, we examined the miR cargo of primary CLL derived exosomes given the importance of miRs in CLL biology.[17, 18] Of significance, we show that CLL exosomes are highly enriched for small RNA species. After the first description of miRs in exosomes, [10] numerous groups have analysed their presence in exosomes.[8] Some miRs were selectively enriched in exosomes, [13, 14] indicating the existence of specific sorting mechanisms [5, 10, 30, 32] that have functional consequences. For example, exosome-contained miRs from cancer cells activate TLR8, and promote release of interleukin-6 and TNFα, in recipient immune cells.[13] We have compared exosomal miRs with CLL cellular miRs using an LNA array consisting of 899 human miRs. The primary cells used in this study represented subsets of CLL with different prognostic variables. In our profiling data we found important miRs, already reported to be of relevance in disease biology, [17, 18, 35] to be equally abundant in cells and exosomes while other miRs such as miR-202-3p, miR-628-3p, and miR-1290 were specifically enriched in exosomes.

In our studies, miR-202-3p was consistently enriched in all exosome samples compared to parental cellular samples. This finding was additionally confirmed by qRT-PCR analysis of selected miR species within exosomes and cellular RNA. These findings are analogous to another study where it was shown that acute myeloid leukaemia (AML) cells generate exosomes that are enriched for miR-146.[40] Similarly, Fabbri et al.,[13] find enrichment of miR-21 and miR-29a in exosomes derived from supernatants of lung cancer cells. The enrichment of miR-202-3p in implies the existence of specific sorting mechanisms within CLL cells that dictate the molecular content of released exosomes.

Deregulation of miR-202-3p is well documented in various tumour types such as cervical, breast, colorectal and gastric cancers.[47, 48] A tumour suppressor role of this miR is suggested by studies showing that it is significantly downregulated in follicular lymphoma compared to follicular hyperplasia.[48, 49] Increased expression of miR-202-3p is generally associated with cell differentiation. With respect to CLL, exosomal release of miR-202-3p from malignant cells into the microenvironment is likely to result in a decrease of its anti-tumorigenic effect. Increased expression of Sufu, a target of miR-202-3p and a negative regulator of Hh signalling, is associated with poor prognosis in CLL.[36] In our study, the expression of Sufu was variable among the CLL cases used but did show a negative correlation with miR-202-3p expression. In addition, we observed a trend for higher levels of expression for Sufu in IgVH unmutated CLL cases, a finding that is in agreement with previous reports of its overexpression in patients with poor prognosis and worse outcomes. Thus, our data suggest that CLL cells specifically package miR-202-3p in order to influence cellular Sufu levels and affect disease behaviour.

In conclusion, we demonstrate that the unique content of secreted exosomes has implications for behaviour of both CLL cells and the cells in the microenvironment. The diverse exosomal cargo of CLL exosomes presents an inherent challenge in assigning a specific functional outcome to the transfer of an individual exosomal miR, mRNA, or protein transcripts. Nevertheless, secretion of exosomes likely has an important role in disease biology and cell behaviour in CLL. In particular, we suggest that the biology surrounding miR-202-3p as a tumour suppressor in CLL is worthy of further investigation. In addition, the role of exosomes and their cargo as potential biomarkers for CLL disease behaviour, and sequestration of specific drugs and indeed monoclonal antibodies (viz. Rituximab) by such microvesicles contributing to drug resistance, may be of relevance and requires further studies.

## Supporting Information

S1 File Table A: Clinical data for CLL cases studied. Figure A: Schematic representation of protocol for harvesting of exosomes. Table B: Antibodies used in this study. Figure B: Primer Sequences. Figure C: Size distribution of total cellular and exosomal RNA. Figure D: Binding site of miR-202-3p in the Sufu 3’UTR. Figure E: Down regulation of Sufu expression in HS-5 cells. Figure F: miR-202-3p expression in exosomes derived from CLL cells and Normal B cells. (DOCX)

S2 File PathwayFinder RT2 Profiler PCR Array. (XLSX)

S3 File LNA miRNA microarray analysis. (XLSX)
