# Supplementary material for: CLL Exosomes Modulate the Transcriptome and Behaviour of Recipient Stromal Cells and Are Selectively Enriched in miR-202-3p
Source: PLoS One. 2015 Oct 28;10(10):e0141429. doi: 10.1371/journal.pone.0141429 (PMC4625016; doi:10.1371/journal.pone.0141429)
Supplement: S1 File — (DOCX) [file pone.0141429.s001.docx]

**Table A**

Clinical data for CLL cases studied. IGVH indicates immunoglobulin heavy chain variable-region; M-mutated, U-Unmutated, and n-not known.

| **Case** | **Age** | **Gender** | **Binet** | **WBC (10^9^/L)** | **IGHV** | **CD38 (%)** | **Karyotype** |
| --- | --- | --- | --- | --- | --- | --- | --- |
| 1 | 73 | f | A | 130 | U | 2 | normal |
| 2 | 75 | f | B | 110 | U | n | n |
| 3 | 62 | m |  | 172 | n | 2 | 13q- |
| 4 | n | m | B | 108 | U | 53 | 11q- |
| 5 | 61 | m | A | 132 | U | 57 | 17p- 13q- |
| 6 | 76 | f | A | 253 | U | 3 | 17p- 13q- |
| 7 | 76 | f | C | 191.5 | U | 2 | normal |
| 8 | 62 | m | B | 134 | n | 25 | 13q- |
| 9 | 73 | m | C | 154 | M | 3 | 13q- |
| 10 | 74 | m | B | 149 | M | 6 | normal |
| 11 | 74 | m | C | 283 | M | 3 | 13q- |
| 12 | 88 | f | A | 150 | M | 0 | n |
| 13 | 54 | m | B | 131 | U | 57 | 17p- 13q- |
| 14 | 61 | m | A | 241 | M | 4 | 17p- 13q- |
| 15 | 69 | m | B | 282 | U | n | 13q- |
| 16 | 76 | m | C | 115 | U | n | 11q- 13q- |
| 17 | 73 | m | B | 236 | n | n | 17p- 13q- |
| 18 | n | m | A | 184.4 | M | n | 11q- 13q- |
| 19 | 70 | f | A | 174.6 | M | 0 | 13q- |
| 20 | 48 | f | A | 116.3 | M | 5 | 13q- |
| 21 | n | m | b | 63.2 | M | 6 | normal |
| 22 | 74 | m | A | 223.2 | U | 60 | normal |
| 23 | 67 | m | A | 178.7 | U | NA | 13q- |
| 24 | 78 | f | n | 150.4 | M | n | n |
| 25 | n | m | C | 126.5 | U | 98 | 11q- 13q- |
| 26 | n | m | A | 226 | U | low | 13q- |
| 27 | 80 | f | B | 126.5 | n | n | 13q- |
| 28 | 56 | f | C | 122 | U | 1 | normal |
| 29 | 67 | m | n | 45.3 | U | 2 | normal |
| 30 | n | m | n | 161.1 | M | n | n |
| 31 | 76 | f | C | n | U | 69 | normal |
| 32 | 78 | f | n | 150.4 | M | n | n |
| 33 | n | m | C | 246 | U | low | 13q- |
| 34 | n | f | A | 161 | n | 36 | 13q- |

**Figure A**

**Schematic representation of protocol for harvesting of exosomes**

Primary CLL cells or cell lines were cultured as described. The culture medium was centrifuged and filtered in a stepwise manner to get rid of cells, microparticles, and debris as indicated. Finally, exosomes were isolated either by further centrifugation or immuno-magnetic isolation.


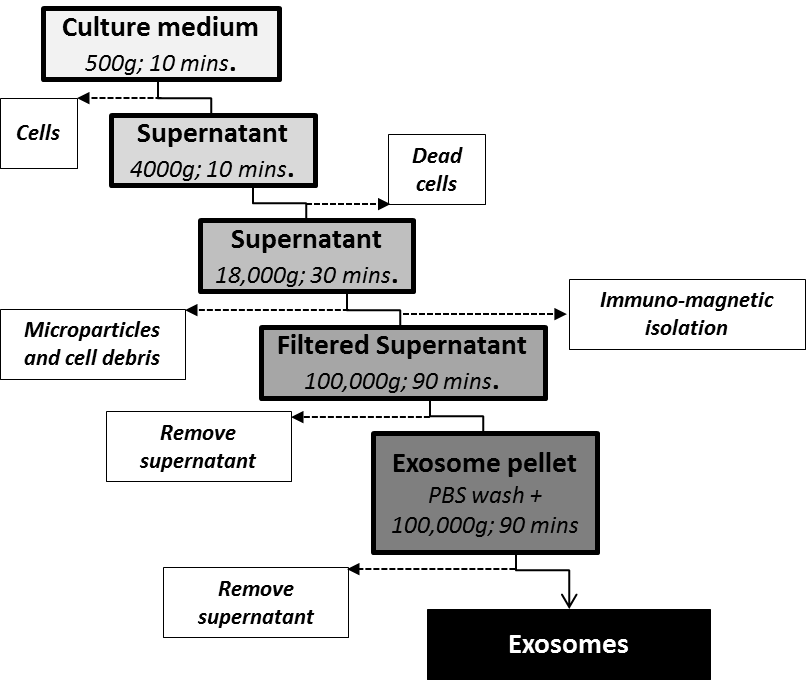


**Table B**

**Antibodies used in this study are as follows**

| Antibody | Source | Catalogue | Manufacturer |
| --- | --- | --- | --- |
| TSG101(clone 5B7) | Mouse mAb, | MCA3515Z | AbD serotec |
| CD81(clone 1D6) | Mouse mAb | ab35026 | Abcam |
| MHC class I (clone EP1395Y) | Rabbit mAb | ab52922 | ab52922 |
| HLA-DR (clone CR3/43) | Mouse mAb | M0775 | Dako Cytomation |
| HLA-ABC (clone G46-2.6) | PE-mouse anti human | 555553 | BD Pharmingen |
| Integrin alpha4(clone 44H6) | Mouse mAb | ab30492 | Abcam |
| Lyn (H6) | Mouse mAb | sc-7274 | Santa Cruz biotechnology |
| gp96/GRP94 (clone 9G10) | Rat | SPA-850 | Stressgen |
| IgM (µ chain specific) | Goat anti-human IgM | 2022-01 | Southern Biotech, |
| Anti-Lamp1 antibody [H4A3] | Mouse mAb | ab25630 | Abcam |
| CD37 (clone M-B371) | Mouse mAb | 555456 | BD Pharmingen |
| CD19 (Clone HIB19 ) | FITC-mouse anti human | 555412 | BD Biosciences |
| Calnexin | Mouse mAb | MAB3126 | Chemicon |
| Anti-Suppressor of Fused antibody [EP1206Y] | Rabbit mAb | ab75835 | Abcam |
| Anti-β-Actin (Clone AC-74) | Mouse mAb | A5316 | Sigma |

**Figure B**

**Primer Sequences**

Primers used in this study purchased from Qiagen. Sufu primers were designed in accordance with published data. [^1^](#_ENREF_1)Sufu primers allowed amplification of both Sufu transcripts.

**Sufu primers:**

Forward: 5′-CGGAGGGGAGAGACCATATT-3′

Reverse: 5′-CACTTGGCACTGACACCACT-3′

**GAPDH primers:**

Forward: 5′-GAAGGTGAAGGTCGGAGTC-3′

Reverse: 5′-GAAGATGGTGATGGGATTT-3′.

1. Hegde GV, Peterson KJ, Emanuel K, et al. Hedgehog-induced survival of B-cell chronic lymphocytic leukemia cells in a stromal cell microenvironment: a potential new therapeutic target. *Mol Cancer Res*. 2008;6(12):1928-1936.

**Figure C**

**Size distribution of total cellular and exosomal RNA**

A representative Bioanalyzer electropherogram shows size distribution of total cellular and exosomal RNA. The percentage of small RNA (<30 nucleotides) was higher in exosomes than in their donor cells. FU: fluorescence units; nt: nucleotides.


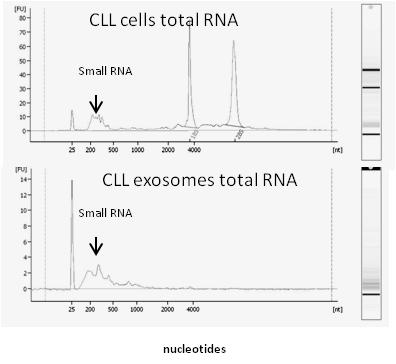


**Figure D**

**Binding site of miR-202-3p in the Sufu 3’UTR**

The 3’ untranslated region (3’UTR) of Sufu with predicted binding sites for miR-202-3p. The figure shows the mature miR-202-3p aligned with both transcripts of Sufu.

**Sufu, transcript variant 1, mRNA (NM_016169):**

**hsa-miR-202-3p 3` AAGGGUACGGGAUAUGGAGA 5`**

**Sufu 2779 5` CAUUCAUGCCCUAUCUUUCA 3`**

**Sufu, transcript variant 2, mRNA (NM_001178133.1):**

**hsa-miR-202-3p 3` AAGGGUACGGGAUAUGGAGA 5`**

**Sufu 1798 5` CUCCCAUGGGCUGUUGCCCA 3`**

**Figure E**

**Down regulation of Sufu expression in HS-5 cells with a synthetic miR-202-3p mimic**

HS-5 cells were transfected with a miR-202-3p mimic or control. After overnight cultures, cell lysates were prepared. Western blots for Sufu and actin were performed as described in the “Materials and Methods” section. As shown, the miR-202-3p mimic suppressed Sufu protein expression. The actin immunoblot confirms equal loading.


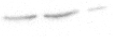


**Actin**

**42kDa**


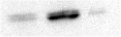


**Sufu**

**54kDa**

**HS-5**

**Exosomes**

**miR-202-3p**

+

+

+

_

**Figure F**

**miR-202-3p expression in exosomes derived from CLL cells and Normal B cells**

Absolute quantification of miR-202-3p expression in exosomes from CLL cells (n=9) and normal B-cells (n=3) was determined by RT-qPCR using the mirVana^TM^ miRNA Reference Panel v9.1. A standard curve was generated for miR-202-3p. Statistical analysis was performed (Mann-Whitney U test) using the SPSS™ v.20 software (p=0.06).
